# Key role of segment IS4 in Cav1.2 inactivation: link between activation and inactivation

**DOI:** 10.1007/s00424-017-2038-3

**Published:** 2017-08-01

**Authors:** Stanislav Andranovits, Stanislav Beyl, Annette Hohaus, Eva Maria Zangerl-Plessl, Eugen Timin, Steffen Hering

**Affiliations:** 1 Department of Pharmacology and Toxicology, University of Vienna, Althanstrasse 14, 1090 Vienna, Austria; 2 Austrian Science Fund (FWF), Haus der Forschung, Sensengasse 1, 1090 Vienna, Austria

**Keywords:** Calcium channel, Voltage sensor, Inactivation, Mutational analysis, Gating, Electrophysiology, Patch clamp, Heart

## Abstract

Inactivation of L-type calcium channel (Cav1.2) is an important determinant of the length of the cardiac action potential. Here, we report a key role of the voltage-sensing segment IS4 in Cav1.2 inactivation. Neutralization of IS4 charges gradually shifted the steady-state inactivation curve on the voltages axis from 5.1 ± 3.7 mV in single point mutant IS4(K1Q) to −26.7 ± 1.3 mV in quadruple mutant IS4(K1Q/R2Q/R3Q/R4Q) compared to wild-type (WT) and accelerated inactivation. The slope factor of the Boltzmann curve of inactivation was decreased from 17.4 ± 3.5 mV (IS4(K1Q)) to 6.2 ± 0.7 mV (IS4(K1Q/R2Q/R3Q/R4Q)). Neutralizations of single or multiple charges in IIS4 and IIIS4 did not significantly affect the time course of inactivation. Neutralization of individual IVS4 charges shifted the inactivation curve between 17.4 ± 1.7 mV (IVS4(R2Q)) and −4.6 ± 1.4 mV (IVS4(R4Q)) on the voltage axis and affected the slope of the inactivation curves (IVS4(R2Q): 10.2 ± 1.2 mV, IVS4(R4Q): 9.7 ± 0.7 mV and IVS4(K5Q): 8.1 ± 0.7 mV vs WT: 14.1 ± 0.8 mV). IS4(K1Q) attenuated while IS4(K1Q/R2Q/R3Q) and IS4(K1Q/R2Q/R4Q/R3Q) enhanced the development of inactivation. Shifts in the voltage dependence of inactivation curves induced by IS4 neutralizations significantly correlated with shifts of the voltage dependence of channel activation (*r* = 0.95, *p* < 0.01) indicating that IS4 movement is not only rate limiting for activation but also initiates inactivation. The paradoxical decrease of the slope factor of the steady-state inactivation and acceleration of inactivation kinetics upon charge neutralization in segment IS4 may reflect the loss of stabilizing interactions of arginines and lysine with surrounding residues.

**Electronic supplementary material:**

The online version of this article (doi:10.1007/s00424-017-2038-3) contains supplementary material, which is available to authorized users.

## Introduction

Calcium entry through voltage-gated calcium channels (Cav) mediates excitation of neuronal and muscle cells and triggers contraction, release of hormones and neurotransmitters, and many other key cellular processes [21, 34, 37]. Membrane depolarization causes Cav not only to open (activation) but induces also channel closure, a process called inactivation. Voltage-dependent inactivation of Cav1.2 develops during the plateau phase of the cardiac action potential (AP) and enables timed repolarization and tuned calcium entry. Loss of inactivation may prolong the cardiac AP several times [3]. The consequences of a failure in inactivation are particular evident from the Timothy syndrome (TS). Prolongation of the cardiac AP in these patients is associated with prolongation of the QT interval, ventricular fibrillation, cardiac arrest, developmental abnormalities, and disorders of the central nervous system [31].

The recently solved structure of Cav1.1 provided the first models for interpretation of the structural basis of opening/closing behavior in the Cav1 family [35, 36]. The α subunit senses the membrane voltage and selectively conducts calcium ions. It is composed of four clockwise arranged homologous domains (I–IV) that are connected by intracellular segments. Each domain contains six transmembrane helices (S1–S6). Helices S5–S6 form the Ca-selective pore and the inner pore gate, whereas the S1–S4 regions represent the voltage sensor domain (VSD). Auxiliary α_2_δ and β subunits tune channel gating and are responsible for the right channel folding and expression [13, 15, 29].

The upward movement of S4 segments in Cav enables conformational rearrangements at the inner S6 helix bundle that leads to pore opening. Mutational analysis and voltage-clamp fluorometry revealed different impacts of S4 segments in Cav1.2 activation [6, 27, 28]. According to Pantazis et al. [27], VSDs II and III contribute ∼85% of the charge necessary for Cav1.2 opening. A mathematical model suggests that upward movement of VSDs II and III is obligatory for Cav1.2 activation [27]. Functional studies on Cav1.2 mutants with completely neutralized IIS4 segments revealed, however, that Cav1.2 activates with similar kinetics as WT and that the effective charge necessary for channel activation is predominantly located in IS4 [6].

The molecular events during Cav1.2 inactivation are less understood. A large number of point mutations in pore lining S6 and adjacent segments of Cav α-subunits has been shown to modulate this process [20, 21, 33]. This includes the Timothy syndrome mutations on Cav1.2 at the cytosolic part of IS6 and a cluster of hydrophobic residues located close to the inner channel mouth on IS6 and IIS6 [14, 22, 24]. Other key inactivation determinants have been identified in intracellular loops [1, 20, 30, 32].

Changes in inactivation caused by S6 mutations on Cav may be very substantial: a 75-fold acceleration of inactivation by a single point mutation was reported for Cav2.1 (M1811Q, [5]) while the Timothy syndrome mutation G402S in Cav1.2 prevents voltage-dependent inactivation almost completely [31].

There is evidence that activation and inactivation in Cav are tightly coupled processes. Hohaus et al. [22] observed a significant correlation between shifts of steady-state activation and inactivation curves caused by six mutations on segment IIS6. An analogical finding was reported by Kudrnac et al. [24] for nine mutations on segments IS6 and IIS6.

The correlation between the positions of the steady-state activation and inactivation curves may reflect two scenarios: (i) voltage-dependent inactivation is triggered by conformational changes in the pore (activation) that are allosterically transmitted to the selectivity filter region adopting a inactivated (non-conducting) conformation [2, 26] or, alternatively, (ii) both processes are coupled via conformational changes in VSDs. Here, we made use of Cav1.2 constructs with fully or partially neutralized charges in S4 segments to elucidate the role of VSDs I–IV in voltage-dependent inactivation.

## Materials and methods

### Mutagenesis

Substitutions in S4 segments of the Cav1.2 α1 subunit (GenBank™ X15539) were introduced using the QuikChange® lightning site-directed mutagenesis kit (Stratagene) with mutagenic primers according to the manufacturer’s instructions. All constructs were checked by restriction site mapping and sequencing.

### Cell culture and transient transfection

Human embryonic kidney (HEK293) tsA-201 cells were grown at 5% CO_2_ and 37 °C to 80% confluence in Dulbecco’s modified Eagle’s/F-12 medium supplemented with 10% (*v*/*v*) fetal calf serum and 100 units/ml penicillin/streptomycin. Cells were split with accutase solution and plated on 35-mm Petri dishes (Falcon) at 60–80% confluence ~24 h before transfection. Subsequently, tsA-201 cells were co-transfected with cDNAs encoding WT or mutant Cav1.2 α1 subunits with auxiliary β3a as well as α2-δ1 [16] subunits and GFP to identify transfected cells.

The transfection of tsA-201 cells was performed using the TurboFect transfection reagent (Thermo Fisher Scientific) following standard protocols. HEK293 cells were used until passage number 26. No variation in channel gating related to different cell passage numbers was observed.

In order to avoid calcium-dependent inactivation, barium ions (20 mM) were used as a charge carrier.

### Ionic current recordings and data acquisition

Barium currents (I_Ba_) through voltage-gated Ca^2+^ channels were recorded at 22–25 °C by patch-clamp [18] using an Axopatch 200A patch clamp amplifier (Axon Instruments, Foster City) 24–48 h after transfection. To avoid calcium-dependent inactivation, barium was used as charge carrier. The extracellular bath solution (in mM: BaCl_2_ 20, MgCl_2_ 1, HEPES 10, choline-Cl 140) was titrated to pH 7.4 with sodium hydroxide. Patch pipettes with resistances of 1 to 4 MΩ were made from borosilicate glass (Clark Electromedical Instruments, UK) and filled with pipette solution (in mM: CsCl 145, MgCl_2_ 3, HEPES 10, EGTA 10), titrated to pH 7.25 with CsOH. All data were digitized using a DIGIDATA 1200 interface (Axon Instruments, Foster City), smoothed by means of a four-pole Bessel filter and saved to disc. One hundred-megasiemen current traces were sampled at 10 kHz and filtered at 5 kHz. Leak currents were subtracted digitally using the average values of scaled leakage currents elicited by a 10-mV hyperpolarizing pulse or electronically by means of an Axopatch 200 amplifier (Axon Instruments, Foster City). Series resistance and offset voltage were routinely compensated for. The pClamp software package (Version 10.0 Axon Instruments, Inc.) was used for data acquisition and preliminary analysis. Microcal Origin 7.0 was used for analysis and curve fitting.

### Analysis of current kinetics

The voltage dependence of activation was determined from I to V curves that were fitted according to the following modified Boltzmann distribution:

$$ I=\frac{G_{\max}\cdot \left(V-{V}_{\mathrm{rev}}\right)}{1+ \exp \left(\frac{V_{\mathrm{m}}-V}{k_{\mathrm{s}}}\right)} $$

where *V* is the membrane potential, *I* is the peak current, *G*_max_ is the maximum membrane conductance, *V*_rev_ is the extrapolated reversal potential, *V*_m_ is the voltage for half-maximal activation, and *k*_s_ is the slope factor. The time course of current inactivation was fitted to a monoexponential function over 3000 ms:

$$ I(t)=A\cdot \exp \left(-t/{\tau}_{\mathrm{inact}}\right)+C $$

where *I*(*t*) is the current at time *t*, *A* is the amplitude coefficient, *τ*_inact_ is the time constant, and C is the steady-state current (see supplemental Fig. S3 for curves fitted to typical current records). Data are given as mean ± S.E.

The voltage dependence of *I*_Ba_ inactivation (inactivation curve) was measured using a double-pulse protocol to account for run-down [19]. The pulse sequence was applied every 60 s from a holding potential of −110 mV, length of conditioning pulse of 3000 ms.

Inactivation curves were drawn according to a Boltzmann equation:

$$ h={h}_{SS}+\frac{1-{h}_{SS}}{1+ \exp \left(\frac{V-{V}_{0.5,\mathrm{inact}}}{k_{\mathrm{inact}}}\right)} $$

where *V* is the membrane potential, *V*_0.5,inact_ is the midpoint voltage, *k*_inact_ is the slope factor, and *h*_SS_ is the fraction of non-inactivating current. Data are given as mean ± S.E. Statistical significance was assessed with the Student’s unpaired *t* test.

### Homology modeling

Sequence identity between the CaV1.1 and CaV1.2 channels is high (56%, according to UniProt). Especially, the VSD and its charged residues are highly conserved (see supplemental Fig. S4 for an alignment of these regions). As a template for modeling, we used the cryo-EM structure of Wu et al. [36]. Wherever necessary, residues important for interactions of the S4 segments as well as the charges in the S4 segments were mutated manually using Swisspdb viewer.

## Results

**Fig. 1:**
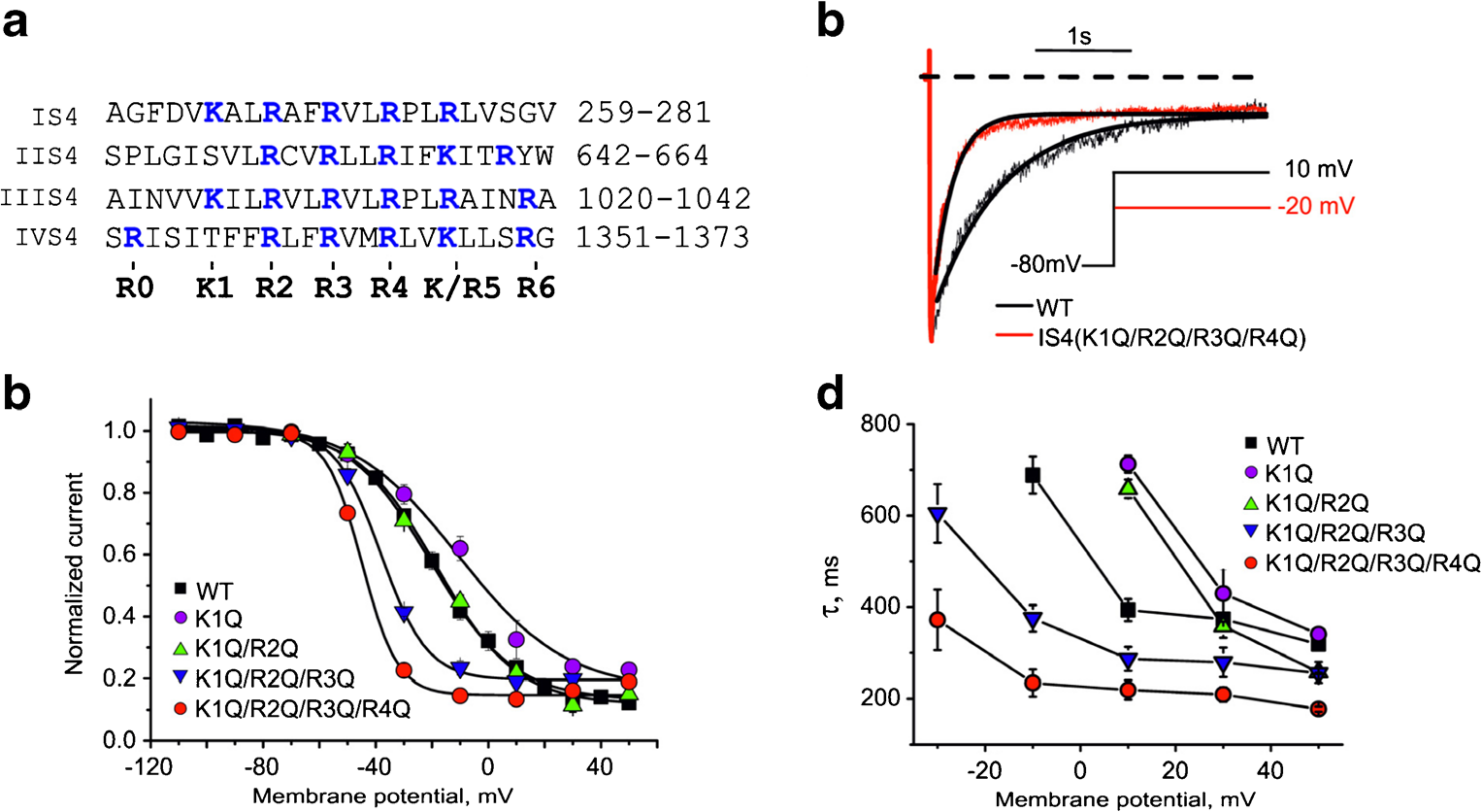
Neutralization of segment IS4 modulates Cav1.2 inactivation. **a** Alignment of Cav1.2 segments IS4-IVS4. Charged residues are *highlighted in blue*. **b** Superimposed typical normalized I_Ba_ through WT and mutant IS4(K1Q/R2Q/R3Q/R4Q). I_Ba_ through WT and quadruple mutant IS4(K1Q/R2Q/R3Q/R4Q) during 3 s depolarizations from −80 mV to the voltages of the maximum of the current-voltage relationship (WT: 10 mV; IS4(K1Q/R2Q/R3Q/R4Q): −20 mV). Note the faster development of inactivation in IS4(K1Q/R2Q/R3Q/R4Q). Current decay was fitted to a monoexponential function yielding time constants of *τ*_inact_(WT) = 393 ± 24 ms and τ_inact_(IS4(K1Q/R2Q/R3Q/R4Q)) = 235 ± 29 ms, respectively (see “Methods”). Solid lines represent the fitted function. **c** Steady-state inactivation curves of WT and the indicated IS4 mutants. Voltages of half-maximal inactivation (*V*_0.5,inact_) where −18.3 ± 1.1 mV (WT), −13.2 ± 3.5 mV (IS4(K1Q)), −20.3 ± 1.2 mV (IS4(K1Q/R2Q)), −38.1 ± 0.8 mV ((IS4(K1Q/R2Q/R3Q)), and −45.0 ± 0.7 mV ((IS4(K1Q/R2Q/R3Q/R4Q)). **d** Inactivation time constants (*τ*_inact_) at different voltages were obtained by fitting the I_Ba_ decay over an interval of 3000 ms by a mono-exponential function. Time constants for WT and the indicated IS4 mutants are plotted as function of the membrane potential

In the present study, we investigate the role of S4 charges in voltage-dependent inactivation of Cav1.2. The Cav1.2 α1 subunit was, therefore, co-expressed with the β_3_ subunit (known to promote channel inactivation, [30]) together with α2-δ. In order to avoid calcium-dependent inactivation, barium ions (20 mM) were used as charge carrier. To elucidate the impact of voltage sensing segments IS4-IVS4 on inactivation, charged arginines or lysines (marked in blue as R and K, Fig. 1a) were step-by-step replaced by glutamines in down-stream direction.

### Neutralization of IS4 charges enhances voltage-dependent inactivation

Figure 1 illustrates the impact of IS4 charges on voltage-dependent inactivation. Replacement of the outermost IS4 charge by glutamine IS4(K1Q) induced a statistically non-significant rightward shift of the steady-state inactivation curve (Δ*V*_0.5,inact_(IS4(K1Q)) = −13.2 ± 3.5 mV vs. Δ*V*_0.5,inact_(WT) = −18.3 ± 1.1 mV, Fig. 1a, Table 1) and neutralization of the two upper charges IS4(K1Q/R2Q) resulted in a left shift compared to IS4(K1Q): *V*_0.5,inact_ (IS4(K1Q/R2Q)) = −20.3 ± 1.2 mV. Neutralization of three IS4(K1Q/R2Q/R3Q) or four charges IS4(K1Q/R2Q/R3Q/R4Q) shifted of the inactivation curve further into the hyperpolarizing direction with midpoint voltages of *V*_0.5,inact_ (IS4(K1Q/R2Q/R3Q)) = −38.1 ± 0.8 mV and *V*_0.5,inact_(IS4(K1Q/R2Q/R3Q/R4Q)) = −45.0 ± 0.7 mV.

**Table 1:** Midpoint voltages and slope factors (*k*) of the activation and inactivation curves

Mutant	*V*_0.5,act_; mV	*K*_act_, mV	*V*_0.5,inact_; mV	*K*_inact_, mV
WT	1.8 ± 0.5 (*n* = 13)	6.6 ± 0.3	−18.3 ± 1.1 (*n* = 10)	14.1 ± 0.8
IS4 mutants				
K1Q	13.2 ± 0.5 (8)	7.2 ± 0.5	−13.2 ± 3.5 (3)	17.4 ± 3.5
K1Q/R2Q	7.1 ± 0.8 (6)	9.4 ± 0.8	−20.3 ± 1.2 (3)	14.7 ± 1.4
K1Q/R2Q/R3Q	−11.9 ± 0.5 (10)	7.3 ± 0.3	−38.1 ± 0.8 (4)	8.2 ± 0.6
K1Q/R2Q/R3Q/R4Q	−20.9 ± 0.3 (10)	8.8 ± 0.3	−45.0 ± 0.7 (6)	6.2 ± 0.7
IIS4 mutants				
R2Q	−7.8 ± 0.7 (3)	8.2 ± 0.7	−22.3 ± 3.6 (3)	13.9 ± 1.9
R6Q	−4.7 ± 0.4 (3)	7.5 ± 0.4	−29.7 ± 1.0 (3)	12.6 ± 0.7
R2Q/R3Q/R4Q/K5Q	−2.2 ± 0.5 (3)	7.0 ± 0.5	−27.8 ± 1.6 (3)	13.8 ± 1.0
R2Q/R3Q/R4Q/K5Q /R6Q	−3.3 ± 1.1 (3)	7.8 ± 1.0	−23.0 ± 1.3 (3)	15.4 ± 1.2
IIIS4 mutants				
K1Q	0.7 ± 0.8 (3)	7.9 ± 0.7	−22.0 ± 3.0 (3)	12.0 ± 1.3
K1Q/R2Q/R3Q/R4Q	−1.8 ± 0.6 (4)	7.7 ± 0.4	−26.3 ± 1.9 (4)	11.3 ± 1.6
IVS4 mutants				
R0Q	5.1 ± 0.3 (5)	7.7 ± 0.3	−17.7 ± 2.5 (5)	13.7 ± 2.3
R2Q	15.0 ± 0.4 (8)	5.9 ± 0.3	−0.9 ± 1.3 (3)	10.2 ± 1.2
R3Q	6.5 ± 0.3 (4)	6.1 ± 0.2	−21.3 ± 1.8 (3)	13.6 ± 1.6
R4Q	0.7 ± 0.6 (3)	5.7 ± 0.4	−22.9 ± 0.9 (3)	9.7 ± 0.7
K5Q	8.9 ± 0.3 (5)	6.5 ± 0.3	−5.6 ± 1.3 (3)	8.1 ± 0.7
R6Q	5.6 ± 0.4 (5)	8.3 ± 0.4	−20.8 ± 2.4 (3)	12.3 ± 1.9

Numbers of experiments are indicated in parentheses

Interestingly, charge neutralization gradually reduced the slope factor (*k*_inact_) of the inactivation curves compared to WT: *k*_inact_(IS4(K1Q/R2Q/R3Q/R4Q)) = 6.2 ± 0.7 mV and *k*_inact_ (IS4(K1Q/R2Q/R3Q)) = 8.2 ± 0.6 mV vs. *k*_inact_ (WT) = 14.1 ± 0.8 mV, see Figs. 1c and 4d. Transfection of cells with cDNA of a construct where all IS4 charges were neutralized did not result in functional channels [6].

Neutralizations of charges in segment IS4 did not only affect the steepness and position of the steady-state inactivation curves but also changed kinetics of the current inactivation. Figure 1b illustrates accelerated inactivation kinetics of IS4(K1Q/R2Q/R3Q/R4Q) compared to WT (see inactivation time constants of this and other mutants in Fig. 1d).

**Fig. 2:**
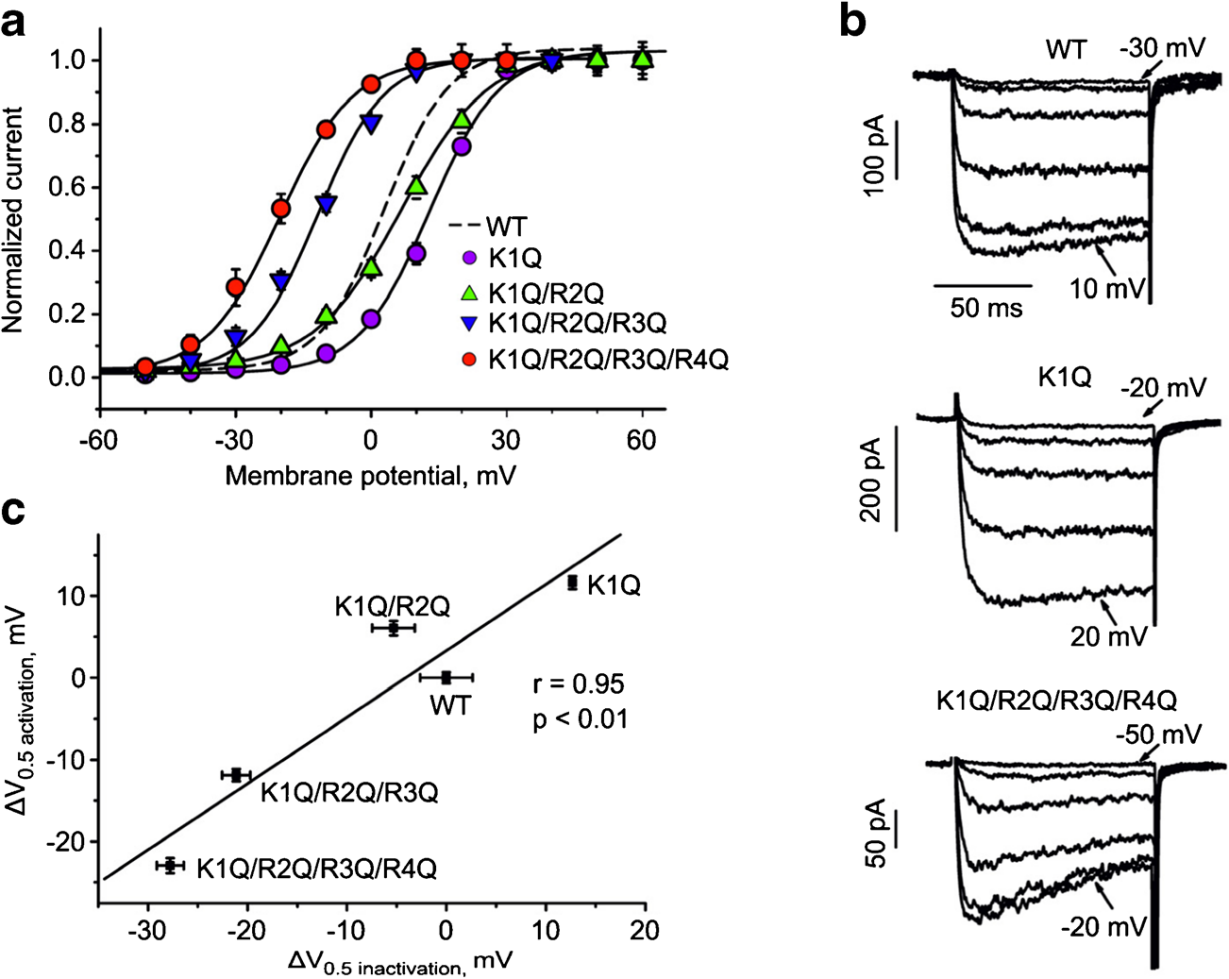
IS4 charge neutralizations cause correlating changes in activation and inactivation. **a** Steady-state activation of Cav1.2 mutants with gradually neutralized IS4 charges. **b** Activation currents of I_Ba_ through WT and mutant channel constructs with partially neutralized S4 segments (IS4(K1Q) and IS4(K1Q/R2Q/R3Q/R4Q)). Currents were recorded by applying a 100-ms pulse from holding potential (−80 mV) with 10 mV steps (WT −30 mV − 10 mV, K1Q −20 mV − 20 mV, K1Q/R2Q/R3Q/R4Q −50 mV − −20 mV). IS4(K1Q/R2Q/R3Q/R4Q) activates at more negative voltages than IS4(K1Q/R2Q/R3Q) and WT while IS4(K1Q) activates at more positive voltages (Table 1). Charge neutralization in IS4 (K1Q/R2Q/R3Q/R4Q) notably accelerates inactivation. Compare typical current recordings of WT (*upper panel*) with mutants K1Q (*middle panel*) and quadruple mutant IS4(K1Q/R2Q/R3Q/R4Q) (*lower panel*). **c**, Correlation between potentials of half-maximal activation (*V*_0.5,act_) and half-maximal inactivation (*V*_0.5,inact_) of IS4 mutants (Table 1). Linear regression analysis (*solid line*) yielded a statistically significant linear correlation (*p* < 0.01) with a correlation coefficient of 0.95

Likewise, observed for the inactivation curve (Fig. 1c), replacement of the outermost charged residue by glutamine shifted the steady-state activation curve significantly rightwards (*V*_0.5,act_(IS4(K1Q)) = 13.2 **±** 0.5 mV, *V*_0.5,act_(WT) = 1.8 ± 0.5 mV, Fig. 2a). Mutants lacking three and four charges activated at significantly more negative voltages (*V*_0.5,act_(IS4(K1Q/R2Q/R3Q)) = −11.9 ± 0.5 mV, *V*_0.5,act_ (IS4(K1Q/R2Q/R3Q/R4Q)) = −20.9 ± 0.3 mV; Table 1). These data are in line with a previous study by Beyl et al. [6] that was performed on Cav1.2 comprising a β_2a_ subunit.

A highly significant (*p* < 0.01) linear correlation between the voltages of half maximal inactivation (*V*_0.5,inact_) and activation (*V*_0.5,act_, Fig. 2c) with *r* = 0.95 was observed.

### Neutralization of IIS4 and IIIS4 charges has less effect on voltage dependence of inactivation

**Fig. 3:**
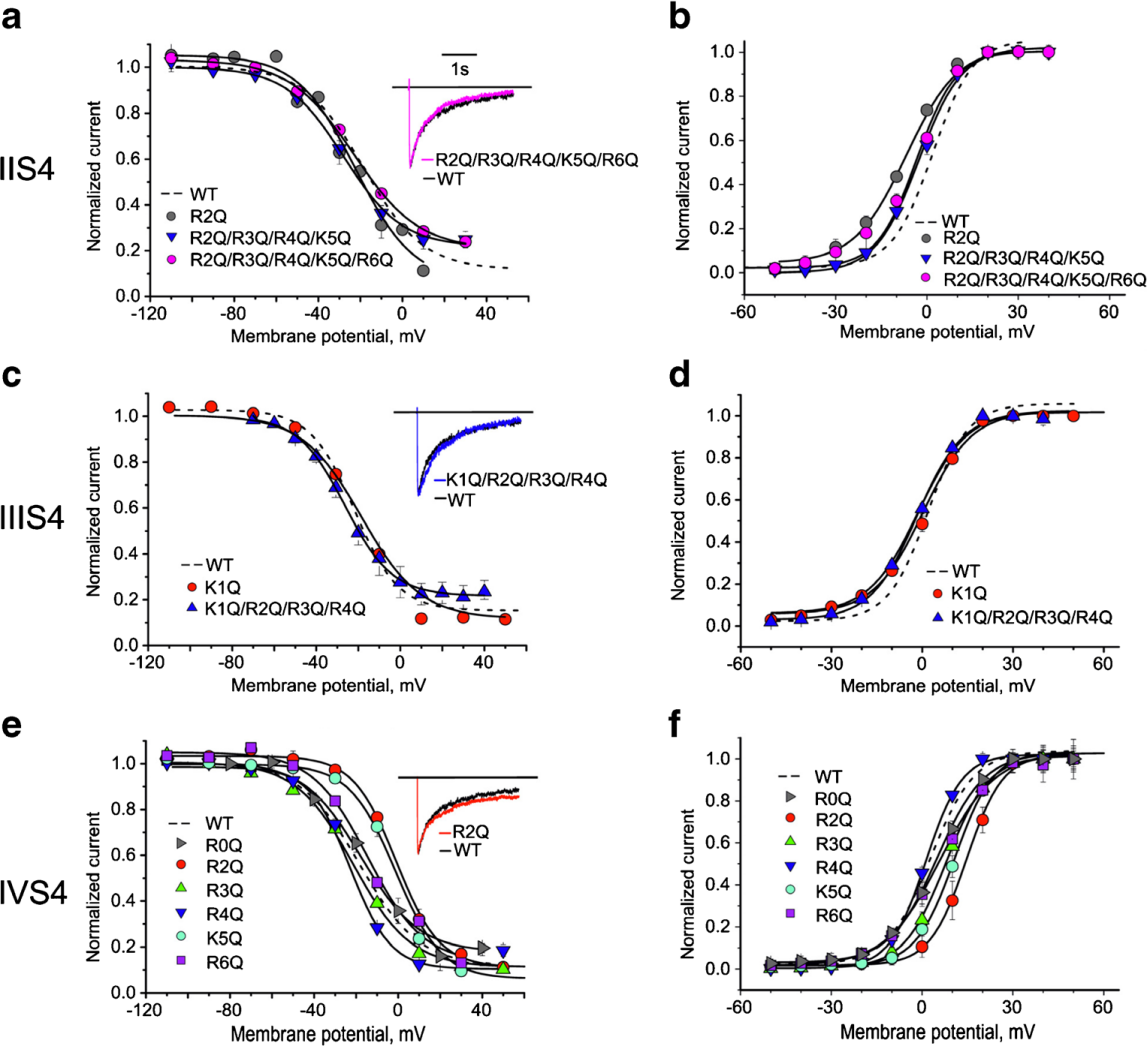
Effect of charge neutralization in IIS4 and IIIS4 on steady-state inactivation and activation. Steady-state inactivation (*left panels*) and activation (*right panels*) curves of WT, IIS4, IIIS4, and IVS4 mutants. Representative barium currents through WT and mutant channels during a 3000-ms depolarization from −80 mV to the peak potential (PP) of the I–V curves are shown in corresponding insets. WT (PP 10 mV); IIS4(R2Q/R3Q/R4Q/K5Q/R6Q) (PP 10 mV); IIIS4(K1Q/R2Q/R3Q/R4Q) (PP 10 mV); IVS4(R2Q) (PP 20 mV)

Figure 3a, c illustrates the effects of charge neutralizations in segments IIS4 and IIIS4 on the inactivation curves. While Cav1.2 tolerates complete removal of all IIS4 charges, expression of functional IIIS4 mutants requires the presence of two charged residues in positions R1041+R1037 (corresponding to R6, R5 in Fig. 1a, [6]). Consequently, the starting points for estimation of the impact of IIS4 and IIIS4 were constructs with completely (IIS4) or partially neutralized (IIIS4) segments.

**Fig. 4:**
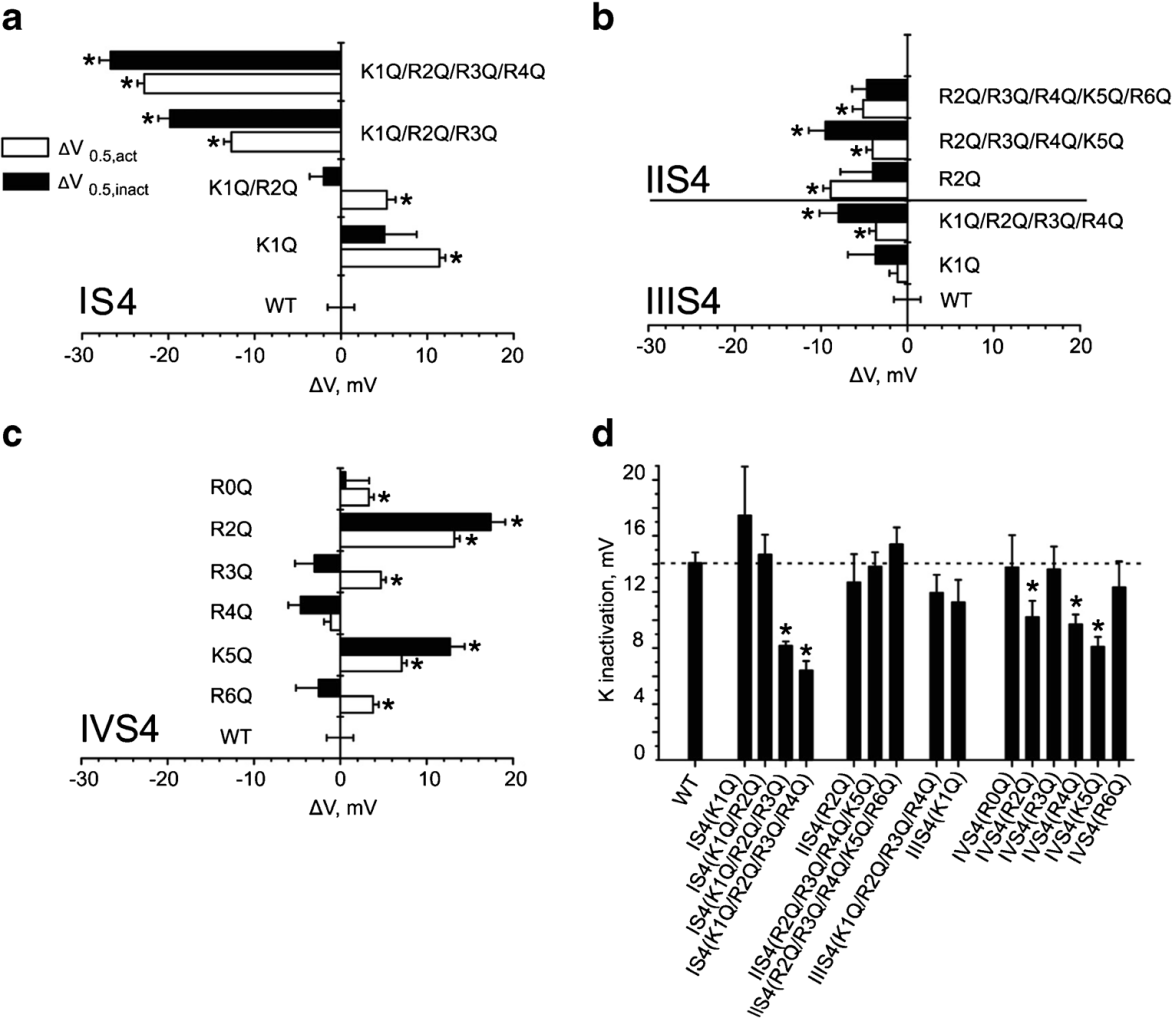
Charge neutralizations in Cav1.2 segments IS4–IVS4 differently affect the position and slope of the inactivation curve. **a**–**c** Shifts of the midpoint voltages (*V*_0.5,inact_ *closed bars*,*V*_0.5,act_ *open bars*) of **a** IS4 , **b** IIS4 and IIIS4, and **c** IVS4-mutants compared to WT. **d** Slope factors of inactivation curves were significantly (*p < 0.05) decreased by charge neutralization in IS4 and IVS4. Strongest changes were observed for mutants IS4((K0Q/R1Q/R2Q) with *k*_inact._ = 8.2 ± 0.6 mV; IS4(K1Q/R2Q/R3Q/R4Q) with *k*_inact._ = 6.2 ± 0.7 mV; IVS4(R2Q) with *k*_inact._ = 10.2 ± 1.2 mV; IVS4(R4Q) with *k*_inact._ = 9.7 ± 0.7 mV and IVS4(K5Q) with *k*_inact._ = 8.1 ± 0.7 mV. Neutralization of either single or combined charge neutralizations in IIS4 and IIIS4 had no significant effect on the slope factors of steady-state inactivation curves (Table 1)

Neither neutralization of all IIS4 charges (IIS4(R2Q/R3Q/R4Q/K5Q /R6Q)) nor partial neutralization of IIS4 or IIIS4 (IIIS4(K1Q/R2Q/R3Q/R4Q)) shifted the midpoints (*V*_0.5,inact_) by more than 10 mV (Figs. 3a, c and 4b).

Representative currents of IIS4(R2Q/R3Q/R4Q/K5Q/R6Q), IIIS4(K1Q/R2Q/R3Q/R4Q), and currents through WT channels at the peak of the current voltage relationship illustrate inactivation kinetics identical to WT (insets in Fig. 3a, c). Similar observations were made for other IIS4 mutants or IIIS4 constructs (Fig. 3a, c). Taken together, substitutions of charged residues in these segments induced only small changes in the voltage-dependence of inactivation compared to the pronounced and gradual effects observed for IS4 mutations on *V*_0.5,inact_ and *k*_inact_ (Fig. 4, see Table 1, see similar small effects in activation curves in Fig. 3b, d).

### Positional specific effects of IVS4 charge neutralization on voltage dependence of inactivation

Combined neutralization of two IVS4 charges prevented formation of functional channel constructs [6]. We have therefore investigated the individual contributions of each of the six IVS4 charges (Fig. 1a). Figure 3e illustrates the corresponding steady-state inactivation curves. Mutations IVS4(R2Q) and IVS4(K5Q) induced significant rightward shifts of inactivation curves (Δ*V*_0.5_,_inact_(IVS4(R2Q)) = −0.9 **±** 1.3 mV; Δ*V*_0.5,inact_(IVS4(K5Q)) = −5.6 ± 1.3 mV) towards more depolarized voltages that were accompanied by shifts of the activation curves in the same direction (Δ*V*_0.5,act._(IVS4(R2Q)) = 15.0 ± 0.4; Δ*V*_0.5,act_(IVS4(K5Q)) = 8.9 ± 0.3, Figs. 3f and 4c, Table 1). Other mutations induced only small or negligible changes. The effects of IVS4 charge neutralizations on the slope factor of the inactivation curve are illustrated in Fig. 4d.

## Discussion

Neutralization of single or multiple charges in S4 segments of voltage-gated ion channels is a productive mean to clarify their role in channel gating (e.g., [4, 9, 17, 25]). Here, we impaired each individual voltage sensor of the Cav1.2 α1 subunit (IS4–IVS4, one at a time) by substituting the charged residues (arginines or lysines) by glutamine. Some of these residues have been shown previously to affect channel activation [6].

We made the following observations: (i) Neutralization of IS4 charges induced pronounced changes in voltage dependence and kinetics of inactivation compared to the lower impacts of IIS4 and IIIS4. (ii) Neutralization of IS4 charges gradually reduced the slope factors of the inactivation curves and accelerated the inactivation kinetics (suggesting a paradoxical enhancement of inactivation). (iii) Shifts of the inactivation curves induced by IS4 neutralization strongly correlated with shifts of the activation curve.

### Key role of IS4 in Cav1.2 inactivation

Pronounced shifts of the inactivation curve upon replacement of IS4 charges by glutamines compared to small changes in IIS4 and IIIS4 mutants highlight the principle role of IS4 in inactivation of Cav1.2 (Figs. 1 and 3). This is particular evident from Fig. 4 illustrating pronounced and gradual shifts of *V*_0.5,inact_ observed for IS4 mutations compared to small changes caused by neutralization of individual or multiple charges in IIS4 and IIIS4.

### Charge neutralization in IS4 enhances voltage sensitivity of inactivation?

The reduction of the slope factors upon charge neutralization (Fig. 4d) compared to irregular and smaller changes in other segments is a further indication of a principal role of IS4 in inactivation (Fig. 1c, Table 1). Formal fitting of these curves to a simple two state Boltzmann distribution yields a paradoxical increase of the effective charge upon neutralization of the physical charge in the VSD.

How can a neutralization of IS4 charges by glutamines enhance voltage sensitivity? Theoretical work of Bezanilla and Villalba-Galea [8] indicates that a simple Boltzmann fit is not applicable for estimation of the effective charge (moving charge times the fraction of the field) if S4 segments move in multiple steps via substates. In such a scenario, the distribution between inactivated and available channels is more shallow than predicted by a two-state Boltzmann function and the slope of the curve is maximal when there are only two states [8].

It is thus tempting to speculate that IS4 in WT channels moves via substates that are transiently stabilized by interactions of arginines (and lysines) with surrounding residues. Replacing these charged residues by neutral glutamines would consequently disrupt these interactions, thereby reducing the number of IS4 substates and decreasing the slope factor of the inactivation curve. Such a hypothesis is also supported by the acceleration of inactivation observed upon stepwise charge IS4 neutralization (Fig. 1d). Hence, removal of IS4 interactions is expected to ease IS4 movements towards an “inactivating” up position.

The cryo-electron microscopy structure of the Ca_v_1.1 complex and the deduced atomic model provide a three-dimensional template for interpretations of interactions of VSD (S4 segments) with surrounding residues. The S1–S3 helices of the VSD in voltage-gated channels contain polar and negatively charged residues that stabilize the positive charges of S4 in different conformations [32–36]. A “gating charge transfer center” (CTC) including a phenylalanine in S2 and negatively charged residues in S2 and S3 has been identified in K_V_ and Na_V_ channels [37, 38, 39]. Hydrogen bonds transiently stabilize charged S4 residues close to the CTC ([39, 40]). The potential interactions to the charged S4 residues are discussed in supplemental materials. Interestingly, in the upstate, IS4 can potentially form up to five salt bridges above the CTC (7 in total) which distinguishes this VSD from domains in segments II–IV (see supplemental material for discussion).

### Role of IVS4 in inactivation gating

In order to get insights into the role of IVS4 in Cav1.2 inactivation we neutralized all six charges individually. Two mutations shifted voltage-dependence of channel gating significantly towards more positive voltages (R2Q: Δ*V*_0.5,inact_ = −0.9 ± 1.3 mV; K5Q: Δ*V*_0.5,inact_ = −5.6 ± 1.3 mV, *p* < 0.05) suggesting that more energy is needed for moving the voltage sensor from its resting to an activated (up) position. Gating of other mutants was not significantly different from WT.

Modeling of the side chain salt-bridge interactions in VSD IV shows only one aspartic acid above the CTC, D1372, which can potentially interact with the positively charged IVS4 residues. In the cryo-EM structure, D1372 interacts with R3; however, during the upward movement of the voltage sensor, interactions with R0 and R2 are conceivable (Fig. 1 in supplemental materials).

### Activation and inactivation of Cav1.2 are linked via segment IS4

There is good evidence that activation of voltage-gated ion channels is associated with conformational changes at the inner helix bundle enabling dissociation of S6 segments and pore opening [10]. Voltage-dependent inactivation is likely to involve structural changes at the outer channel mouth at the selectivity filter [11–13, 23].

Evidence for a close link between the activation and inactivation gates in Cav1.2 comes from multiple observations that perturbations of the activation gate (inner helix bundle) usually affect voltage-gated inactivation. Thus, significant correlations between the shifts of the activation and inactivation curves caused by mutations on segments IS6–IVS6 suggest that conformational changes at the inner helical bundle are coupled to the inactivation gate [14, 22, 24].

Here, we present first evidence that structural changes in voltage-sensing domains of Cav1.2 affect channel inactivation and activation. This is particularly evident from IS4 mutations causing the largest shifts of the voltage dependence of activation and inactivation (Figs. 1c and 2a, c). We speculate that the up movement of IS4 is not only rate limiting for pore opening but also triggers conformational changes leading to inactivation.

## Conclusions and outlook

Taken together, we identified a dominant role of segment IS4 in voltage-dependent inactivation of Cav1.2. Impairing its function by charge neutralization had the largest and regular (charge-dependent) effects on voltage-dependent inactivation compared to equivalent (quadruple) charge neutralizations in segments IIS4 and IIIS4 causing only small gating perturbations (Figs. 1, 3, and 4). Furthermore, stepwise neutralization of IS4 charges induced a parallel and gradual reduction of the slope factor of the inactivation curve (Fig. 4d). Enhancement of voltage-dependent inactivation upon IS4 neutralization was also evident from the accelerated time course of current decay (Fig. 1b, d).

We speculate that mutations of the positively charged residues in IS4 eliminate “stabilizing” interactions with neighboring segments thereby reducing the number of IS4 substates. Channel activation may, thus, be enabled by a small “pore releasing” S4 movements [6, 7] while inactivation involves an up movement via multiple intermediate states. The nature of IS4 substates (corresponding interactions with neighboring segments) has yet to be investigated (Fig. S1).

The role of IVS4 in activation and inactivation gating remains unclear and requires additional studies.

## Electronic supplementary material

ESM 1 (DOCX 1714 kb)
