# Supplementary material for: Key role of segment IS4 in Cav1.2 inactivation: link between activation and inactivation
Source: Pflugers Arch. 2017 Aug 1;469(11):1485–93. doi: 10.1007/s00424-017-2038-3 (PMC5629230; doi:10.1007/s00424-017-2038-3)
Supplement: Supplementary file 1 — (DOCX 1714 kb) [file 424_2017_2038_MOESM1_ESM.docx]

**Key role of segment IS4 in Cav1.2 inactivation:
Link between activation and inactivation**

**Stanislav Andranovits, Stanislav Beyl, Annette Hohaus, Eva Maria Zangerl-Plessl, Eugen Timin, Steffen Hering***

From the Department of Pharmacology and Toxicology, University of Vienna,

Althanstrasse 14, 1090 Vienna, Austria

##### SUPPLEMENTARY MATERIAL

The CTC (Fig. S1) facilitates the movement of the positively charged S4 residues in response to changes in membrane voltage [[9](#_ENREF_9)]. The cryo-EM structure of Wu et al. [[11](#_ENREF_11)], [[10](#_ENREF_10)] appears to represent the activated (up) state of a voltage sensor domain, and in every S4 segment, 4 charged residues are on the extra-cellular site of the CTC [[6](#_ENREF_6)], [[7](#_ENREF_7)], [[1](#_ENREF_1)]. The pore domain of the structure represents, however, the closed state. Most recently, a new cryo-EM structure from the eukaryotic Na_V_PaS channel with the VSDs in different VSD-states by Shen et al. [[8](#_ENREF_8)] was published and discussed in context with the CaV1.1 structure.

In the CaV1.1 cryo-EM structure, only 14 salt-bridge interactions (within 4Å ([[5](#_ENREF_5)])) in the VSDs are seen. However, modelling of the side chain conformations using SwissPDB Viewer [[4](#_ENREF_4)] of this structure shows that all together 21 salt-bridge interaction can occur (see supplemental Fig. S2) . Interestingly, all four positively charged residues of IS4 (K1, R2, R3 and R4) above the CTC can form salt-bridge interactions with negatively charged residues from the IS2 (K1 with D180, R2 with E190, R3 with E190 and E193 and R4 with E193 and R5 with E203 (see Fig. S2)). This also implicates, that upon depolarization, the charged residues on the IS4 segment interact sequentially with these acidic residues on S2 segments [[3](#_ENREF_3)], [[2](#_ENREF_2)], [[12](#_ENREF_12)] , which supports the hypothesis that IS4 activation occurs via sub-states.

***Supplemental Figures***


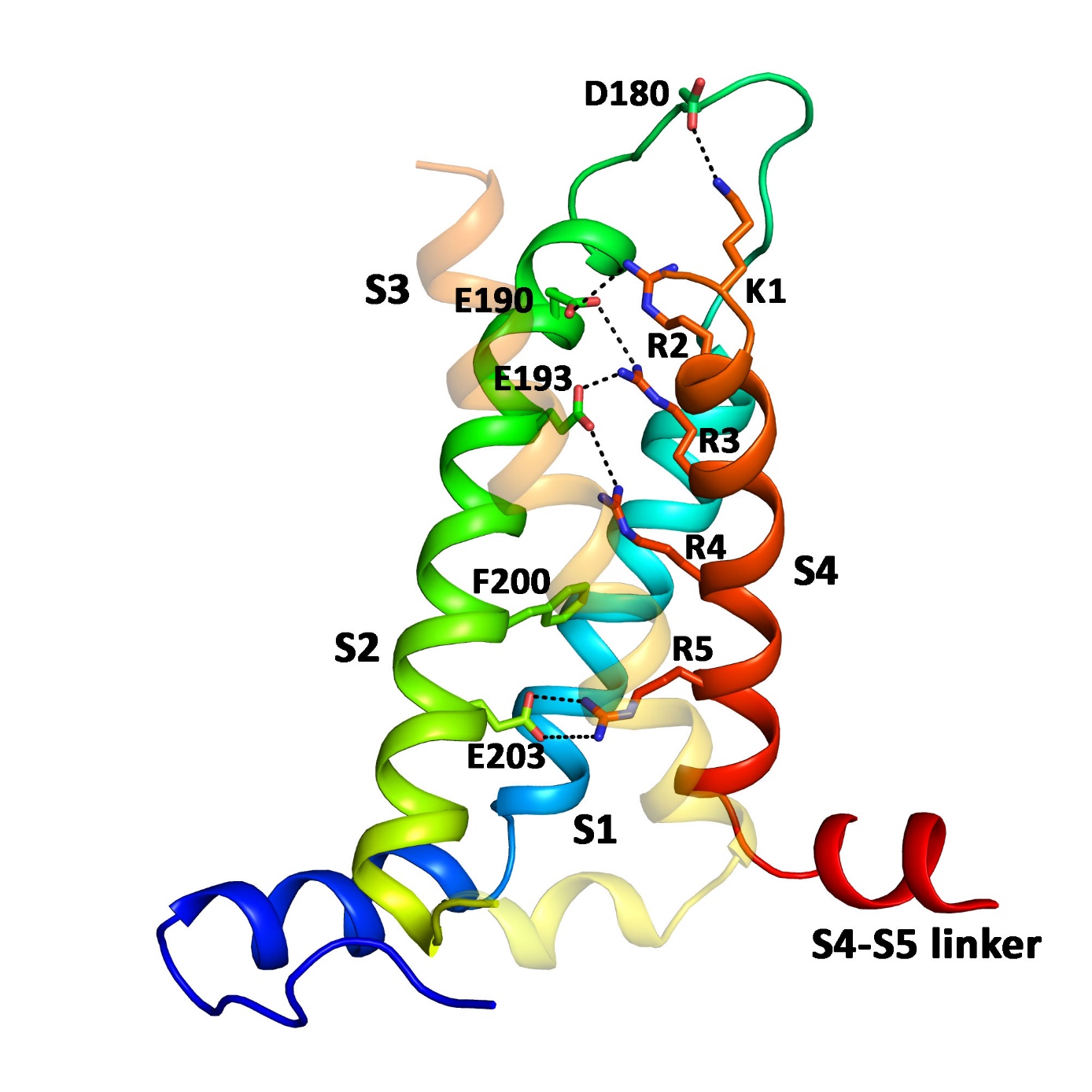


**Fig. S1** Potential interactions of the S4 charged residues in VSD I in the CaV1.2 homology model. S1, S2, S3 and S4 presented as cartoon in blue, green, yellow and red, respectively. Charged residues represented as sticks. Charge interaction possibilities are highlighted as black dash lines. For clarity reasons, the S3 is slightly transparent.


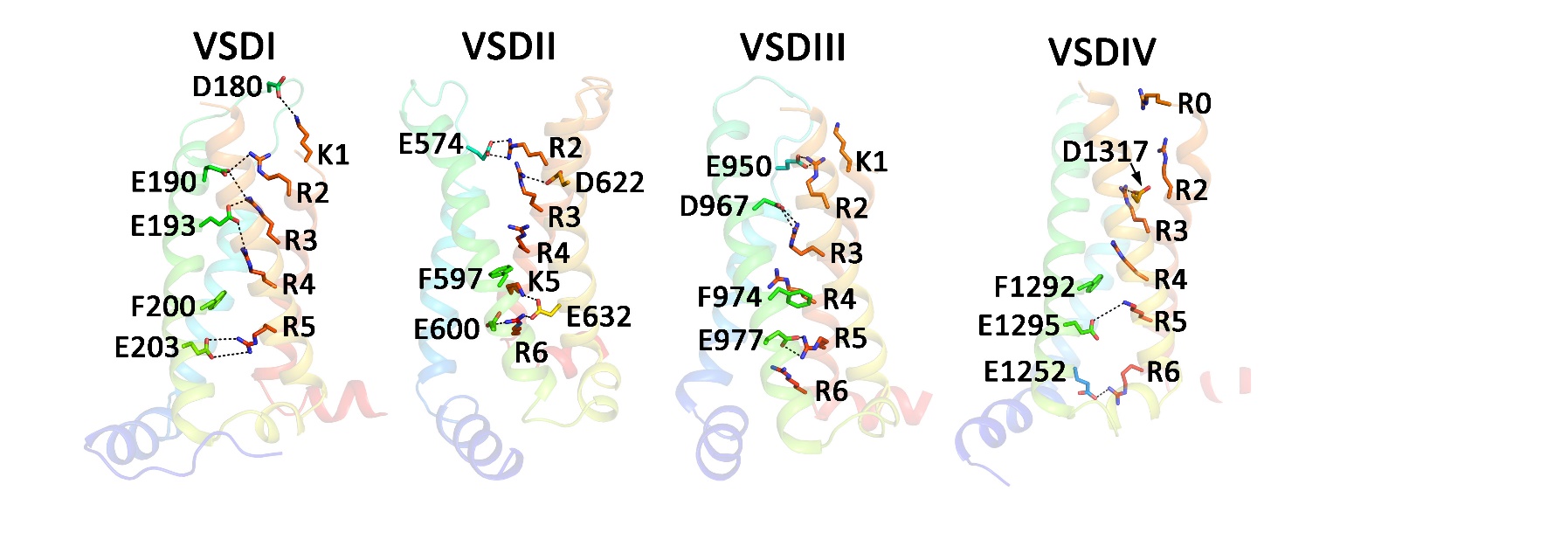


**Fig. S2** VSDI-IV interactions of the positively charged S4 residues in the produced homology model. Cartoon representation of the S1-S4 helices. Charged residues that can interact are shown as sticks. Interaction possibilities are shown as dashed lines. Maximum distance for interactions was 4Å.


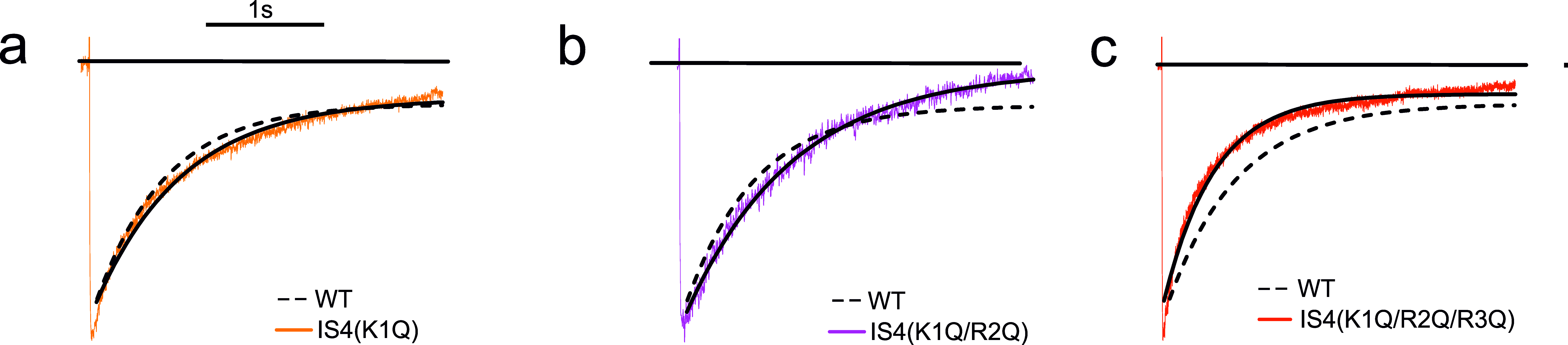


**Fig. S3** Decays of typical barium currents were fitted to mono-exponential curve during voltage steps from -80 mV to PP of the current voltage-relationships of WT (PP: 10mV, broken line), IS4(K1Q) (PP: 20 mV, **a**) IS4(K1Q/R2Q) (PP: 20 mV, **b**), IS4(K1Q/R2Q/R3Q) (PP: -10 mV, **c**). The function fitted to WT current decay is indicated as broken line in **a-c**.


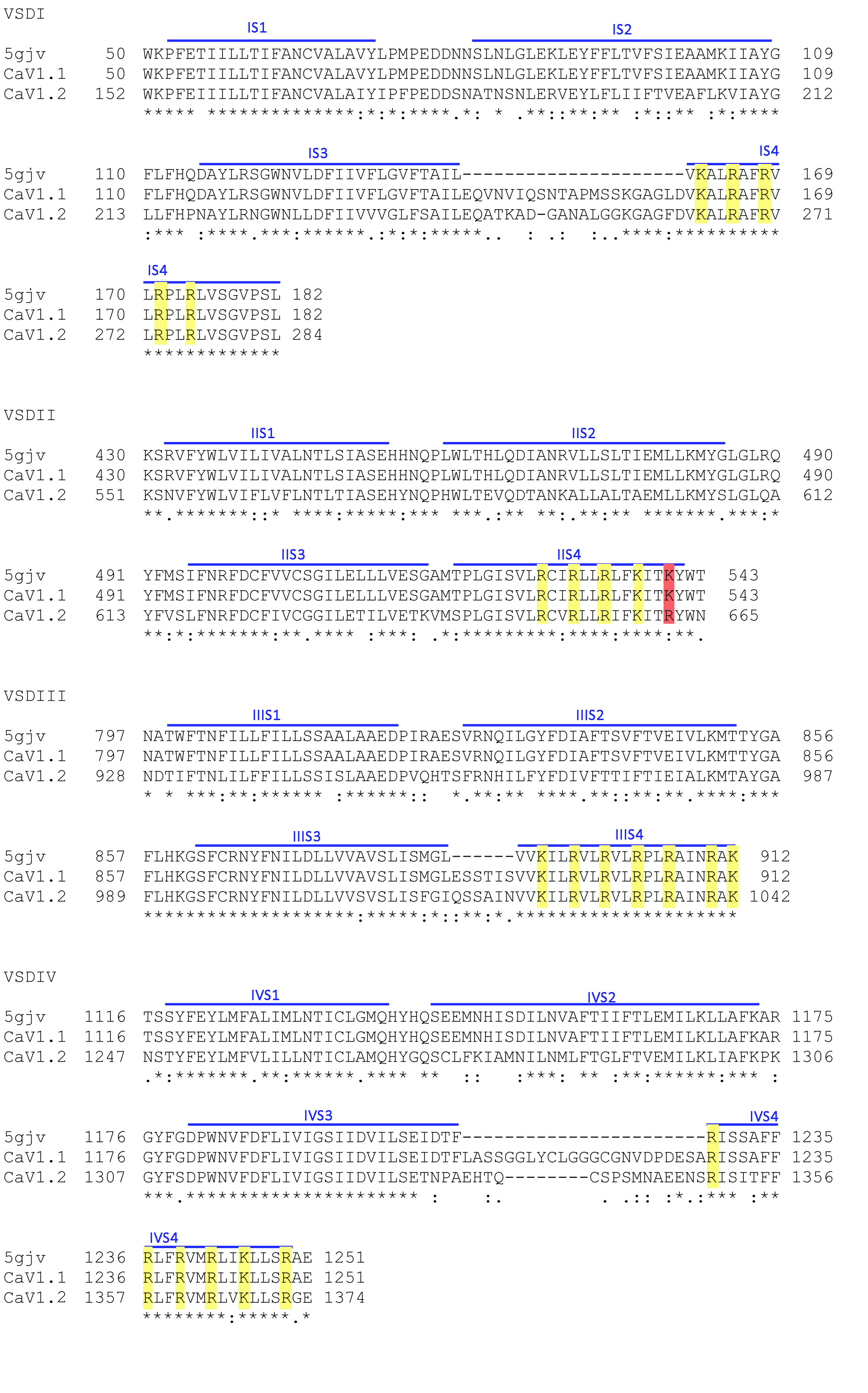


Fig. S4 Sequence alignment of all four VSDs of the CaV1.1 crystal structure (pdb code: 5gjv), the CaV1.1 sequence of the rabbit (pdb code: P07293) and the CaV1.2 rabbit sequence (pdb code: P15381). Highlighted in yellow are the positively charged amino acids in the all S4 segments and in red the only non-conserved charged amino acid in IIS4. On top of the lines in blue we highlighted the helices according to the crystal structure (5gjv).

**REFERENCES**

1. Aggarwal SK, MacKinnon R (1996) Contribution of the S4 segment to gating charge in the Shaker K+ channel. Neuron 16:1169-1177

2. DeCaen PG, Yarov-Yarovoy V, Sharp EM, Scheuer T, Catterall WA (2009) Sequential formation of ion pairs during activation of a sodium channel voltage sensor. Proceedings of the National Academy of Sciences of the United States of America 106:22498-22503. doi:10.1073/pnas.0912307106

3. DeCaen PG, Yarov-Yarovoy V, Zhao Y, Scheuer T, Catterall WA (2008) Disulfide locking a sodium channel voltage sensor reveals ion pair formation during activation. Proceedings of the National Academy of Sciences of the United States of America 105:15142-15147. doi:10.1073/pnas.0806486105

4. Guex N, Peitsch MC (1997) SWISS-MODEL and the Swiss-PdbViewer: an environment for comparative protein modeling. Electrophoresis 18:2714-2723. doi:10.1002/elps.1150181505

5. Kumar S, Nussinov R (2002) Close-range electrostatic interactions in proteins. Chembiochem : a European journal of chemical biology 3:604-617. doi:10.1002/1439-7633(20020703)3:7<604::AID-CBIC604>3.0.CO;2-X

6. Schoppa NE, McCormack K, Tanouye MA, Sigworth FJ (1992) The size of gating charge in wild-type and mutant Shaker potassium channels. Science 255:1712-1715

7. Seoh SA, Sigg D, Papazian DM, Bezanilla F (1996) Voltage-sensing residues in the S2 and S4 segments of the Shaker K+ channel. Neuron 16:1159-1167

8. Shen H, Zhou Q, Pan X, Li Z, Wu J, Yan N (2017) Structure of a eukaryotic voltage-gated sodium channel at near-atomic resolution. Science 355. doi:10.1126/science.aal4326

9. Tao X, Lee A, Limapichat W, Dougherty DA, MacKinnon R (2010) A gating charge transfer center in voltage sensors. Science 328:67-73. doi:10.1126/science.1185954

10. Wu J, Yan Z, Li Z, Qian X, Lu S, Dong M, Zhou Q, Yan N (2016) Structure of the voltage-gated calcium channel Ca(v)1.1 at 3.6 A resolution. Nature 537:191-196. doi:10.1038/nature19321

11. Wu J, Yan Z, Li Z, Yan C, Lu S, Dong M, Yan N (2015) Structure of the voltage-gated calcium channel Cav1.1 complex. Science 350:aad2395. doi:10.1126/science.aad2395

12. Zhang X, Ren W, DeCaen P, Yan C, Tao X, Tang L, Wang J, Hasegawa K, Kumasaka T, He J, Wang J, Clapham DE, Yan N (2012) Crystal structure of an orthologue of the NaChBac voltage-gated sodium channel. Nature 486:130-134. doi:10.1038/nature11054
